# Preparation and Characterization of an Amphipathic Magnetic Nanosphere

**DOI:** 10.1155/2014/976145

**Published:** 2014-03-05

**Authors:** Yongsheng Ji, Ruihong Lv, Zhigang Xu, Chuande Zhao, Haixia Zhang

**Affiliations:** ^1^College of Pharmacy, Henan University of Traditional Chinese Medicine, Zhengzhou 450000, China; ^2^Key Laboratory of Nonferrous Metal Chemistry and Resources Utilization of Gansu Province, Lanzhou University, Lanzhou 730000, China

## Abstract

The amphipathic magnetic nanospheres were synthesized using C_8_ and polyethylene glycol as ligands. Their morphology, structure, and composition were characterized by transmission electron microscope, Fourier transform infrared, and elementary analysis. The prepared materials presented uniform sphere with size distribution about 200 nm. The magnetic characteristics of magnetic nanomaterials were measured by vibrating sample magnetometer. The target products had a saturation magnetization value of 50 emu g^−1^ and superparamagnetism. The adsorption capability was also studied by static tests, and the material was applied to enrich benzenesulfonamide from calf serum. The results exhibited that the C_8_-PEG phase owned better adsorption capability, biocompatible property, and dispersivity in aqueous samples.

## 1. Introduction

Magnetic nanoparticles (MNPs) have been the hot topic of scientific research. The reports not only were concerned with their fundamental properties, but also involved the promotion of their characteristics and real applications [1]. The researches concerning the promotion of MNPs' properties focused on the chemical modification onto the surface of MNPs in order to accommodate different aim of application. MNPs have been applied extensively in various fields, for example, magnetic storage media, bioseparation [2–7], drug delivery [8], biomolecular sensors [9], magnetic resonance imaging [10], jet printing [11], and so on.

Recently, magnetic nanoparticles coupled to magnetic carrier technology (MCT) have also been used in solid-phase extraction (SPE), especially for analyzing environmental and biological samples. Song et al. [12], Zhao et al. [13, 14], and Li et al. [15] utilized bare Fe_3_O_4_ or silicon-coated Fe_3_O_4_ as extracting sorbent to enrich organic pollutants. Yantasee et al. [16] took thiol-modified MNPs to remove metallic ion from water. Qi et al. [17] immobilized Zr^4+^ ions on the surface of MNPs for selective enrichment of the phosphopeptides. Liu et al. [18] and Yao et al. [19] prepared magnetic C_18_ and C_8_ microspheres to extract polycyclic aromatic hydrocarbons (PAHs) from water and polypeptide from serum, respectively. Zhang et al. [20] reported the application of magnetic molecularly imprinted polymers (MIP) in solid or semisolid samples for trace analysis of triazines and tetracycline antibiotics. Niu et al. [21] prepared carbon-encapsulated MNPs for isolation of organic pollutants. The magnetic MIP with bisphenol A (BPA) as a template was also prepared and applied to extract BPA from environmental water and milk samples [22]. The extraction can be easily achieved by dispersing the magnetic sorbents to sample solution, and the sorbents can be effortlessly isolated from the mixture by an external magnet. Compared to common SPE with nanomaterials, the magnetic SPE not only can build a controllable rebinding process and allow magnetic separation to replace the centrifugation and filtration step in a convenient and economical way, but can also avoid troubles of packing SPE column and the time-consuming process of loading large volume samples. It has been successfully employed in rapid pretreatment of large volume samples.

The dispersivity and compatibility of sorbents used in magnetic SPE are the key factors for their application. Several attempts have been done to fabricate better magnetic sorbents. MNPs using *β*-cyclodextrin (*β*-CD) as ligand were manufactured, and they showed superior dispersivity in water [23]. Hu et al. [24] improved the biocompatibility of magnetic MIP by combining SPE with liquid-liquid extraction (LLE). Li et al. [25] synthesized amphoteric magnetic microspheres owning hydrophilicity and hydrophobicity. But they could not take consideration to dispersivity and biocompatibility. However, the biocompatibility was necessary for the samples containing biomolecules. C_8_ and C_18_ materials are the classical sorbents of SPE. Polyethylene glycol (PEG) due to hydrophilic and biocompatible properties has been used as ligand to prepare various functional materials [1, 26]. It would be a promising sorbent to simultaneously bond C_8_ and PEG onto the surface of MNPs.

In the present work, magnetic nanospheres possessing hydrophilic, hydrophobic, and biocompatible properties were produced with C_8_ and PEG as ligands. The obtained products were applied to enrich benzenesulfonamide from calf serum.

## 2. Experimental

### 2.1. Chemicals and Materials

Sodium acetate (NaAc), triethylamine (TEA), polyethylene glycol (PEG), ethylene glycol, and ferric chloride (FeCl_3_ · 6H_2_O) were obtained from Tianjin Chemicals Corporation (Tianjin, China). Tetraethoxysilane (TEOS) and octyltrimethoxysilane (OCS) were purchased from Fluka (Switzerland). These chemicals were all of analytical grade. Sulfamerazine (SMR) was purchased from Alfa Aesar (Karlsruhe, Germany; >98.5%). Acetonitrile and methanol of chromatography grade were purchased from Dima Technology (Richmond Hill, USA). All other reagents were of analytical reagent grade and the purified water by Milli-Q system was used throughout the experiments.

### 2.2. Preparation of Magnetic Nanoparticles

**Figure 1:**
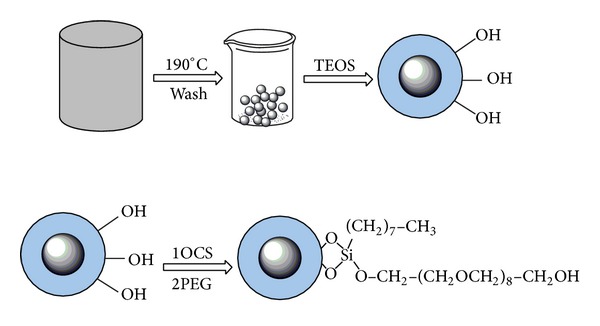
Preparation protocol of the magnetic nanospheres.

The preparation protocol of magnetic nanospheres is shown in Figure 1.

Ferric chloride (2.7 g, 10 mmol) was dissolved in ethylene glycol (80 mL) to form a clear solution, followed by the addition of NaAc (7.2 g) and polyethylene glycol (MW800, 1.0 g). After ultrasonication for 10 min, the mixture was stirred vigorously for 30 min. Then, it was sealed in a teflon-lined stainless-steel autoclave (100 mL capacity). The autoclave was maintained at 190°C for 10 h and then cooled to room temperature. The black products (Fe_3_O_4_) were washed several times with ethanol and dried at 60°C for 12 h.

The obtained magnetic nanoparticles were encapsulated into silica beds as follows [26]: 1.0 g Fe_3_O_4_ was suspended in 200 mL ethanol, then ammonia (25%, 30 mL), water (30 mL), and TEOS (3 mL) were added sequentially into the generated solution. After degassed, the mixture was stirred vigorously for 2 h at 50°C under nitrogen gas protection. The magnetic particles were separated from the resulting solution by a magnet and the supernatant was discarded. The target materials were rinsed with ethanol to remove excess reactants, neutralized with HCl (0.1 mol L^−1^), and washed with water. The obtained materials (Fe_3_O_4_@SiO_2_) were dried under vacuum at 80°C for 12 h.

One gram of Fe_3_O_4_@SiO_2_, 60 mL of toluene, 1 mL of OCS, and 0.5 mL of TEA were added successively to a flask. The mixture was refluxed under nitrogen gas protection and stirring for 10 h. After cooling, the magnetic particles were separated from the mixture by a magnet and the C_8_ bonded phase was rinsed with toluene. The obtained products were dispersed again to 60 mL of toluene. 1 mL of PEG (MW400) and 0.5 mL of TEA were added successively to the mixture. After refluxing and stirring for 10 h, the C_8_-PEG bonded materials were obtained and rinsed with toluene and ethanol. The products were dried under vacuum at 80°C for 12 h.

### 2.3. Characterization

The obtained products were characterized with JEM1200EX transmission electron microscope (TEM) (Tokyo, Japan) and Nicolet Nexus 670 Fourier transform infrared (FTIR) (MN, USA) spectrometer. Elementary analysis (EA) was performed on a Vario-EL analyzer (Elementar, Germany). Magnetic properties were measured using a vibrating sample magnetometer (VSM) (Lakeshore 7304, USA).

### 2.4. Adsorption Tests

To measure adsorption capacity, 20 mg of C_8_-PEG was added into 10 mL SMR solution with desired concentration. The mixture was shaken for 12 h at room temperature to facilitate adsorption of SMR onto C_8_-PEG phase. After the magnetic sorbents were isolated by an external magnetic field, SMR of the supernate was determined by high performance liquid chromatography (HPLC). The chromatographic system consisted of Varian 210 high performance liquid chromatographic pump (CA, USA), 325 UV-Vis detector, and Varian Star Chromatographic workstation. An analytical reversed-phase C_18_ column (5 *μ*m, 4.5 × 250 mm, Dima Technology Richmond Hill, USA) was used. The mobile phase was a mixture of acetonitrile and water (60 : 40, *v/v*) with flow rate of 0.8 mL min⁡^−1^ and the detection was carried out at 263 nm. The same procedure was performed for the C_8_ bonded material. All tests were conducted in triplicate.

### 2.5. Application in Real Samples

The samples of calf serum with 10 *μ*g mL^−1^ SMR were diluted to 4, 2, and 1 *μ*g mL^−1^, respectively. 20 mg of C_8_-PEG activated by methanol and water was added to 10 mL of diluted samples, after vortex and standing for 5 min, and then the sorbents were isolated by an external magnet. The sorbents were washed by water and hexane and then eluted by 3 mL of acetonitrile containing 1% acetic acid. At last, the elution was analyzed by HPLC-UV.

## 3. Results and Discussion

### 3.1. Characterization

#### 3.1.1. FTIR

**Figure 2:**
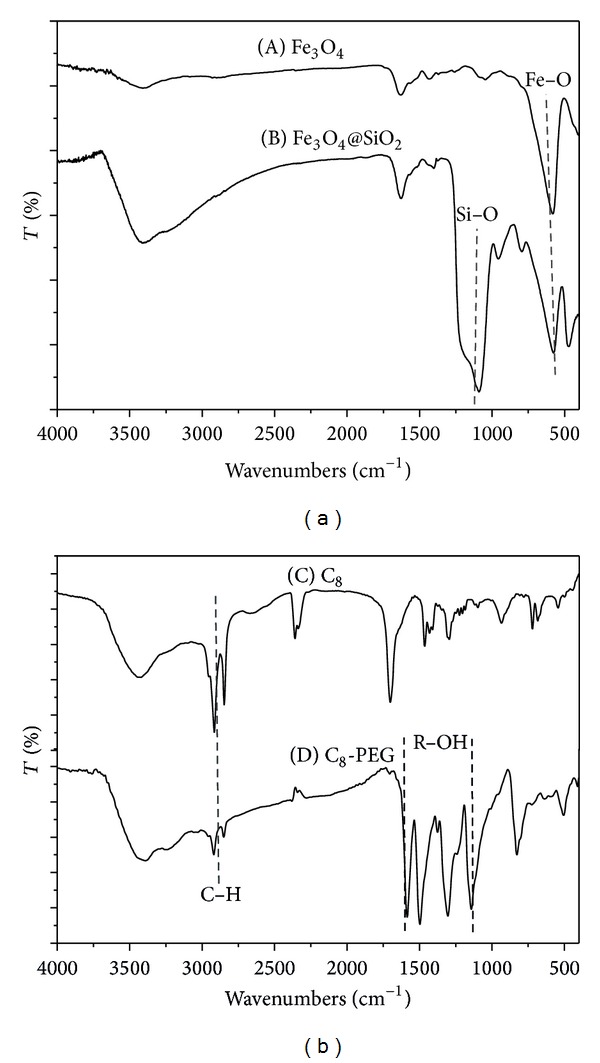
FTIR of (A) Fe_3_O_4_, (B) Fe_3_O_4_@SiO_2_, (C) C_8_, and (D) C_8_-PEG.

All products were measured by FTIR spectrometry step by step, and the results are listed in Figure 2. IR spectra provided clear evidence for the surface modification.  Figure 2(a) displays the IR spectrum of the bare magnetic particles and the characteristic band of Fe_3_O_4_ appeared at about 580 cm^−1^. The unique Si-O absorption band was observed from 1000 to 1100 cm^−1^ (Figure 2(b)), indicating that silica coating was successful on the magnetite surface. In spectra of C_8_ and C_8_-PEG bonded particles a typical band of C–H stretch was about 2900 cm^−1^, and the adsorption bands between 1100 and 1600 cm^−1^ were related closely to R–OH, suggesting that the C_8_ and C_8_-PEG phase were prepared successfully.

#### 3.1.2. EA

Elemental analysis was employed to measure the composition of C_8_ and C_8_-PEG phase products, and the results are listed in Table 1. Comparing C_8_-PEG to C_8_ phase, the mass fraction of C and H increased, confirming that PEG was successfully bonded onto the surface of magnetic nanospheres. However, it cannot ensure precisely the component of C and H on the surface of magnetic nanospheres based on the data of elemental analysis.

#### 3.1.3. TEM

**Figure 3:**
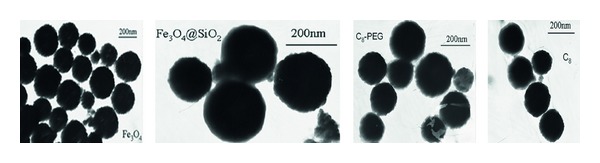
TEM of Fe_3_O_4_, Fe_3_O_4_@SiO_2_, C_8_, and C_8_-PEG.

Their morphological feature of all products was observed by TEM in Figure 3. As can be seen, most of the obtained Fe_3_O_4_ nanospheres exhibited regularly spherical shape with size distribution about 200 nm. After the modification of silica, the magnetic nanospheres showed core-shell structure, and the thickness of shell was estimated to be about 20–30 nm. No significant differences were observed between C_8_ and C_8_-PEG phase.

#### 3.1.4. VSM

**Figure 4:**
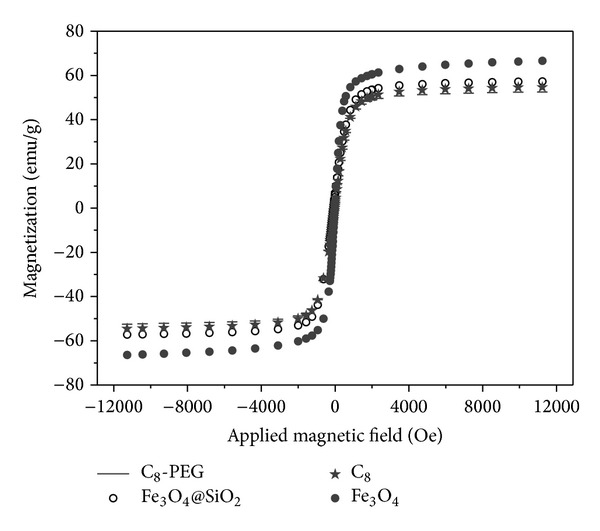
VSM magnetization curves of Fe_3_O_4_, Fe_3_O_4_@SiO_2_, C_8_, and C_8_-PEG.

It is vitally important for MCT that the sorbents should possess sufficient magnetic and superparamagnetism property. VSM was employed to characterize magnetic properties of the obtained magnetic materials, and the VSM magnetization curves are shown in Figure 4. All magnetization curves were uniformly symmetric, confirming that the prepared products presented superparamagnetism. It can be seen that the saturation magnetization value of Fe_3_O_4_, Fe_3_O_4_@SiO_2_, C_8_, and C_8_-PEG decreased, respectively, due to the increase of nonmagnetic density. However, the saturation magnetization value of C_8_-PEG was 50 emu g^−1^, which indicated that C_8_-PEG phase materials possessed superior magnetic property.

#### 3.1.5. Dispersivity

**Figure 5:**
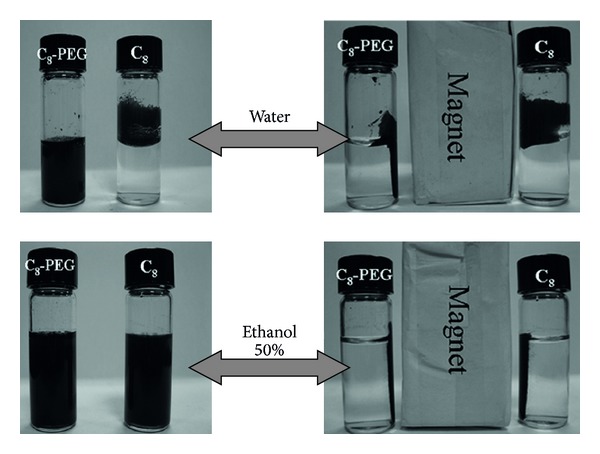
Comparison of C_8_ and C_8_-PEG dispersed in water.

The dispersivity was investigated in order to evaluate the difference of C_8_ and C_8_-PEG. As shown in Figure 5, 20 mg of magnetic materials was dispersed to 2 mL of water or 50% ethanol solution. From Figure 5(a), the left photograph displays that C_8_ and C_8_-PEG were dispersed to water by sonication, and the right photograph shows that the sorbents were collected by magnet after 10 s. The dispersivity of C_8_-PEG in water was visibly better than C_8_. Figure 5(b) illustrates that C_8_ and C_8_-PEG were dispersed to 50% ethanol solution, and no evident differences of dispersivity were observed. The results confirmed that the magnetic material can be separated easily from solution by an external magnet and C_8_-PEG phase presented superior property to C_8_.

### 3.2. Adsorption Tests

**Figure 6:**
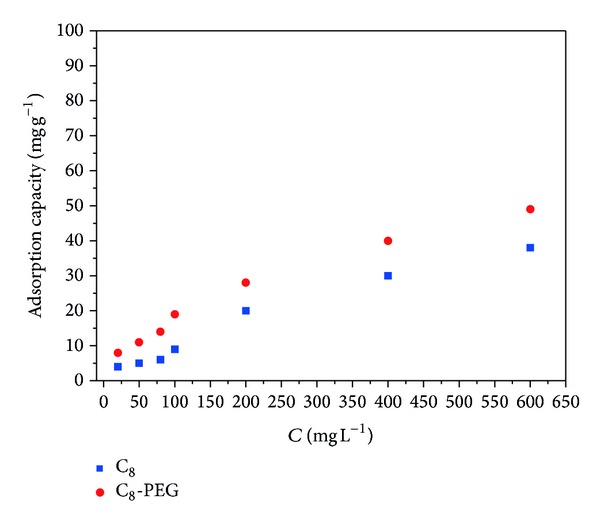
Static adsorption of SMR with C_8_ and C_8_-PEG.

The adsorption capability of C_8_ and C_8_-PEG was researched by static tests with SMR as the target compound. As shown in Figure 6, the capacity of C_8_-PEG was about two times of C_8_ with the concentration of SMR in the range 20–100 mg L^−1^, and the differences decreased with the increase of SMR concentration. The differences were caused by their different dispersivity in sample solution. The sample solution used in static tests was obtained by diluting the methanol solution of SMR 1000 mg L^−1^. C_8_-PEG was better dispersed in sample solution than C_8_ when the SMR concentration was lower, and the differences of dispersivity between C_8_ and C_8_-PEG reduced while the concentration of SMR was higher. Furthermore, the hydrogen bond between O or OH of PEG and amine of SMR also contributed to the differences. The tests revealed that C_8_-PEG presented superior adsorption property to C_8_ for liquid samples.

### 3.3. Application in Real Samples

**Figure 7:**
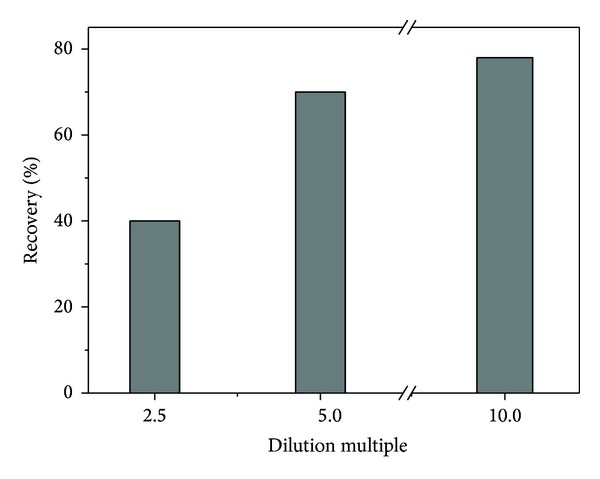
Recoveries of SMR from calf serum treated by C_8_-PEG.

In order to study the feasibility of C_8_-PEG in the real application, it was applied to enrich SMR from calf serum and the spiked recoveries were investigated. As shown in Figure 7, the recoveries rose by 40% while the dilution ratio of the spiked samples (SMR, 10 *μ*g mL^−1^) increased from 2.5 to 10. Under the condition of 10-times dilution, C_8_-PEG could achieve better recovery without other pretreatments, confirming that it can reduce effectively the matrix effect, for example, the influences of protein, polypeptide, fat, and so on. The results demonstrated that C_8_-PEG owning biocompatibility can be applied in the pretreatment of biological samples.

## 4. Conclusion

The novel magnetic nano-spheres were prepared using C_8_ and polyethylene glycol as ligands. The prepared materials presented uniform sphere with size distribution about 200 nm and superparamagnetism. The results of tests proved that C_8_-PEG owned better dispersivity in aqueous samples, adsorption capability, and biocompatible property. C_8_-PEG was used to enrich SMR from calf serum and the spiked recoveries were 80%. C_8_-PEG performed superior potential in biological samples.

## Figures and Tables

**Table 1:** Elemental analysis of C_8 _and C_8_-PEG phase.

Solid phase	C (%)	H (%)
C_8_	2.523	0.882
C_8_-PEG	2.990	1.182
